# Transmembranous and enchondral osteogenesis in transplants of rat limb buds cultivated in serum‐ and protein‐free culture medium

**DOI:** 10.1111/ahe.12835

**Published:** 2022-07-11

**Authors:** Marta Himelreich Perić, Vedrana Mužić‐Radović, Tihana Marić, Floriana Bulić‐Jakuš, Gordana Jurić‐Lekić, Marta Takahashi, Nino Sinčić, Davor Ježek, Ana Katušić‐Bojanac

**Affiliations:** ^1^ Center of Excellence in Reproductive and Regenerative Medicine School of Medicine Zagreb Croatia; ^2^ Department of Biology School of Medicine Zagreb Croatia; ^3^ Hospital for Medical Rehabilitation of the Heart and Lung Diseases and Rheumatism ‐Thalassotherapia Opatija Opatija Croatia; ^4^ Department of Histology and Embryology School of Medicine Zagreb Croatia; ^5^ Department of Communicology Catholic University of Croatia Zagreb Croatia

**Keywords:** in vitro, limb bud, osteogenesis, serum‐free, transplantation

## Abstract

Cartilage differentiates in rat limb buds cultivated in a chemically defined protein‐free medium in the same manner as in the richer serum‐supplemented medium. We aimed to investigate the remaining differentiation potential of pre‐cultivated limb buds by subsequent transplantation in vivo. Rat front (FLBs) and hind‐limb buds (HLBs) were isolated from Fischer rat dams at the 14th gestation day (GD 14) and cultivated at the air‐liquid interface in Eagle's Minimum Essential Medium (MEM) alone; with 5 μM of 5‐azacytidine (5azaC) or with rat serum (1:1). Overall growth was measured seven times during the culture by an ocular micrometre. After 14 days, explants were transplanted under the kidney capsule of adult males. Growth of limb buds was significantly lower in all limb buds cultivated in MEM than in those cultivated with serum. In MEM with 5azaC, growth of LBs was significantly lower only on day 3 of culture. Afterwards, it was higher throughout the culture period, although a statistically significant difference was assessed only for HLBs. In transplants, mixed structures developed with the differentiated transmembranous bone, cartilage with enchondral ossification, bone‐marrow, sebaceous gland, and hair that have never been found in vitro. Nerves differentiated only in transplants precultivated in the serum‐supplemented medium. We conclude that pre‐cultivation of LBs in a chemically defined protein‐free medium does not restrict osteogenesis and formation of epidermal appendages but is restrictive for neural tissue. These results are important for understanding limb development and regenerative medicine strategies.

## 1 INTRODUCTION

Skeletal injuries and diseases represent a significant health issue. As comprehensively reviewed (Ehnert et al., 2020), advanced model systems are necessary to research possible treatments and underlying developmental mechanisms. Among such are ex vivo culture models of limb buds or mandibles being closest to the developmental in vivo situation while preserving the surrounding soft tissue (Ehnert et al., 2020; Muzic et al., 2013; Paradis et al., 2019; Smith et al., 2010). In addition, research on human and animal in vitro cell cultures aiming to produce tissues for transplantation has revealed the superiority of three‐dimensional in vitro models over the two‐dimensional cell culture models because the latter lack tissue interactions necessary for optimal differentiation results (Nürnberger et al., 2013; Tortelli & Cancedda, 2009).

Embryological processes occurring during limb development are the focus of “developmental engineering” that aims to repair bone defects resulting from trauma, congenital abnormalities, cancer, or other diseases. Most bone tissue engineering strategies mimic embryonal intramembranous osteogenesis, where the bone is produced by mesenchymal stem cells, but poor perfusion leading to avascular necrosis and core degradation is delaying its clinical translation. Recapitulating the embryonic processes of endochondral ossification from the primary cartilage, which is more resilient to the avascular environment, may have a better effect (Fu et al., 2021).

Our previous research showed that rat limb buds from the 13th gestational day (GD 13) could continue developing in a natural three‐dimensional organ‐culture model at the air‐liquid interface ex vivo. After a two‐week culture in vitro, cartilage and epidermis differentiate well in a serum‐supplemented medium (Muzic et al., 2013). Moreover, in this organ‐culture model, GD 13 and GD 14 limb buds differentiate cartilage equally in the serum‐supplemented and chemically defined serum‐ and protein‐free medium (Mužić Radović et al., 2022). Chemically defined media in experiments of growing cartilage and bone cells from different sources are superior to the serum‐ or platelet‐lysate‐supplemented media because those supplements are undefined and of a variable composition that may interfere with the research results (Xu et al., 2020). Moreover, the frequently used foetal bovine serum might have a negative impact on necessary gene expression for the cartilage matrix synthesis (Wu et al., 2013). Additionally, xeno‐free media formulations devoid of animal serum should be included when cultures are prepared for ready‐to‐use clinical applications (Palamà et al., 2020).

Normal limb development requires strict gene expression regulation provided by genetic and epigenetic mechanisms such as DNA methylation, histone modifications, and RNA interference. A specific global DNA methylation dynamics of repetitive sequences (SINE) associated with chondrogenesis was shown in the limb bud developmental system we use. A rise in global DNA methylation was associated with less differentiated organ primordia in cartilage development (Mužić Radović et al., 2022). 5‐azacytidine (5azaC), a DNA demethylating epigenetic therapeutic (Voso et al., 2015) that is known to activate gene expression, significantly impaired the growth of limb buds cultivated in the model used previously (Muzic et al., 2013). 5azaC was also implicated in global DNA demethylation of limb buds associated with congenital anomalies of limbs after in vivo application (Sobočan et al., 2019). It was shown that 5azaC exerts distinct effects associated with different phases of mouse cartilage formation in micromass cultures, namely by reducing cartilage‐specific gene expression and cartilage formation when applied during the early stages of chondrogenesis and a stimulatory effect when added to differentiated chondrocytes (Vágó et al., 2021). An age‐related decline in the proliferation of primary human adipose‐derived mesenchymal stem cells was reversed by incubation with 5azaC, inducing proliferation and improving the osteogenic differentiation potential (Yan et al., 2014).

In vitro conditions strive to mimic the environmental complexity necessary for normal chondrogenesis and osteogenesis, but they are still inferior to the in vivo environment in providing developmental requirements. Additionally, pluripotent stem cell cultures in vitro, differentiating towards bone or cartilage, may still harbour remnants of stem cells that may form teratoma tumours once transplanted in vitro. Therefore, the results of in vitro cultures should be finally verified in vivo (Bulic‐Jakus et al., 2016; Ehnert et al., 2020). The most used in vivo techniques are topic transplantation of bone or cartilage grafts grown in vitro or ectopic subcutaneous transplantation (Shiota et al., 1990). Our results obtained previously regarding transplantation under the kidney capsule of in vitro cultivated gastrulating embryos‐proper showed that this site is optimal for the continuation of chondrogenesis, osteogenesis, neurogenesis, and development of skin appendages. In vivo results also reflected results obtained by previous cultivation in vitro (Belovari et al., 2001; Bulic‐Jakus et al., 2001).

We have previously shown that terminal differentiation of the cartilage in rat limb buds cultivated in vitro can proceed in the simple chemically defined protein‐free medium (Mužić Radović et al., 2022). Our present research aims to compare growth in vitro and osteogenesis in syngeneic transplants in vivo of rat limb buds pre‐cultivated in the simple chemically defined protein‐free medium and the serum‐supplemented medium.

## 2 MATERIAL AND METHODS

### 2.1 Ethical statement

The research was conducted with the permission of the Ethics Committee of the Medical Faculty of the University of Zagreb (No. 04‐76/2006‐9) and according to the laws and regulations of the Republic of Croatia and EU directive 2010. on animal experimentation.

### 2.2 Experimental animals

Inbred males and females of three months old albino rat Fisher strain were used from the Department of Biology of the Medical Faculty of the University of Zagreb. Dams were placed in a cage with males overnight for mating. The next morning, the presence of sperm in the vaginal smear was marked as the first day of pregnancy (GD 1).

### 2.3 Isolation of limb buds

Pregnant dams were euthanized on the GD 14 with a lethal combination of 48 mg/kg of anaesthetic Xylapan (xylazine) with sedative, muscle relaxing, and analgesic effects and 300 mg/kg Narketan 10 (ketamine) with a narcotic effect. Under the dissecting microscope, deciduas with embryos were isolated from the uterine horns, and limb buds were cut off from isolated embryos.

### 2.4 Rat serum obtainment

Rat serum was obtained from male Fisher rats. Animals were anaesthetized by an intraperitoneal injection of a mixture of Xylapan (12 mg/kg body weight) and Narketan 10 (75 mg/kg body weight) solutions. Under aseptic conditions, the abdominal cavity was opened, and blood was drawn from the abdominal aorta that euthanized animals. The blood was immediately centrifuged at 2500 g for 15 min. After centrifugation, the serum was inactivated by heating at 56 °C for 30 min and sterilized by filtration through a 0.22 μm Millipore filter, aliquoted, and stored at −20 °C.

### 2.5 Culture in vitro

Isolated limb buds (explants) were cultivated in disposable organ culture vessels (Falcon 3037) with a central well. A stainless‐steel grid was placed in the well and covered with a piece of porous lens paper. Explants were placed on the paper with a braking pipette, and that day was marked as the 0‐day of culture. The cultivation media, poured into the well to moisten the lens paper, were Eagle's minimum essential medium with Hank's balanced salt solution (MEM), MEM with rat serum supplement (1:1), and MEM with 5 μM 5‐azacytidine (A2385; Sigma Aldrich). Media were changed every other day. The explants were grown for 14 days in an incubator at the air‐liquid boundary in 5 % CO_2_ and 95 % air at 37 °C in a water‐humidifying atmosphere.

### 2.6 The overall growth of explants

The growth of ellipsoidal explants of limb buds was monitored by measuring major and minor diameters using an ocular micrometre placed in a dissecting microscope at a magnification of × 1.6. Diameters were measured at plating, concomitant with the changes of media, and on the last day of culture, altogether seven times. The obtained measurements were included in the formula for the ellipse area (*A* = area): *A* = π × larger diameter × smaller diameter/4. The areas calculated at the individual days of culture were normalized by dividing them with A0 values obtained at the plating of limb buds. For day zero, the calculated area A/A0 was 1 (Muzic et al., 2013).

### 2.7 Transplantation

14‐day‐old explants were subsequently transplanted under the kidney capsule of adult male Fischer rats. Explants were carefully separated from the paper under a binocular dissecting microscope and washed in MEM. To anaesthetize the animal, an intraperitoneal injection of Xylapan (12 mg/kg body weight) and Narketan 10 (75 mg/kg body weight) mixture was used. In the right lumbar region, through an incision of the skin and muscle, the kidney was non‐traumatically recovered. Then the kidney capsule was gently incised, avoiding bleeding, and a small “pocket” was made between the capsule and the parenchyma where the explant was placed. The abdominal wall and skin were closed with Michel clamps. Animals were housed in different cages according to the type of in vitro pretreatment (4 animals per experiment) and maintained for 14 days under standard conditions with daily veterinary control.

### 2.8 Histology and immunohistochemistry

After 14 days spent in vivo, transplants were fixed for classical histological or immunohistochemical (IHC) processing in the mild Sainte Marie fixative (96 % alcohol and 1 % glacial acetic acid) for 24 h. After paraffin embedding, paraffin blocks were serially sectioned (5 μm). After deparaffinization, routine haematoxylin–eosin or Safranin O staining was done, and sections were then rehydrated and covered (Mužić Radović et al., 2022).

For immunohistochemistry (IHC), reagents from DAKO were used according to the manufacturer's instructions, i.e., primary antibody monoclonal mouse‐anti‐proliferating cell nuclear antigen (PCNA), Clone PC10 (M0879) (1:100), followed by Labelled streptavidin‐biotin kit, (K0609) Dakocytomation, LSAB®2 System–HRP, and Streptavidin/HRP (P0397). A nonspecific antibody was used for negative controls (No. V 1617 mouse IgG1, DAKO). The antigen retrieval was done by heating in the ChemMateTM Target Retrieval Solution (x 10) solution (S2031; DAKO, Glostrup, Denmark).

### 2.9 DNA isolation

Six limb bud samples per group were deparaffinized in xylene (2 × 5 min) and then incubated in 100 %, 95 %, and 70 % ethanol, each for 3 minutes. DNA was isolated in TE buffer pH 9 with 0.1 mg/ml of Proteinase K and 0.25 % of Nonidet P40 at 56 °C (24 h). Samples were heated for 10 min (95 °C) to inactivate Proteinase K and spun. The supernatant was frozen (−20 °C). DNA quality was assessed, and concentrations were measured with the NanoDrop ND‐2000 spectrophotometer (NanoDrop Technologies).

### 2.10 Bisulphite conversion and polymerase chain reaction

A quantity of 1000 ng of unpurified isolated genomic DNA was used for bisulphite conversion by EpiTect Plus DNA Bisulfite Kit (#59124; Qiagen) with a clean‐up step. PyroMark PCR Kit (#978703; Qiagen) was used for PCR amplification at 95 °C for 2 min, 43 °C for 90 s, and 72 °C for 60 s for 40 cycles. PCR forward primer was 5′‐GGGTTGGGGATTTAG‐3′, and biotinylated reverse primer: 5’‐AACCCAAAACCTTA‐3′.

### 2.11 Pyrosequencing

Pyromark Q24 Advanced System using PyroMark Q24 CpG Advanced Reagents (#970922; Qiagen) was used for the pyrosequencing reaction, following the recommendation by Qiagen. 5′‐GGGGATTTAGTTTAGTGGT‐3′ was the sequencing primer for the ID element of the rat (Kim et al., 2007). DNA methylation data were obtained and analysed by the PyroMark Q24 Advanced Software. Quality control of the data was determined during the pyrosequencing run as described before (Mužić Radović et al., 2022).

### 2.12 Statistical analysis

D'Agostino and Pearson Omnibus tests were used to determine the normality of data distribution. Student's *t*‐test was used for growth values, and Mann–Whitney *U*‐test was used for DNA‐methylation data. Results with *p* values <0.05 were considered statistically significant.

## 3 RESULTS

### 3.1 Growth of limb buds cultivated in chemically defined protein‐free medium

**FIGURE 1:**
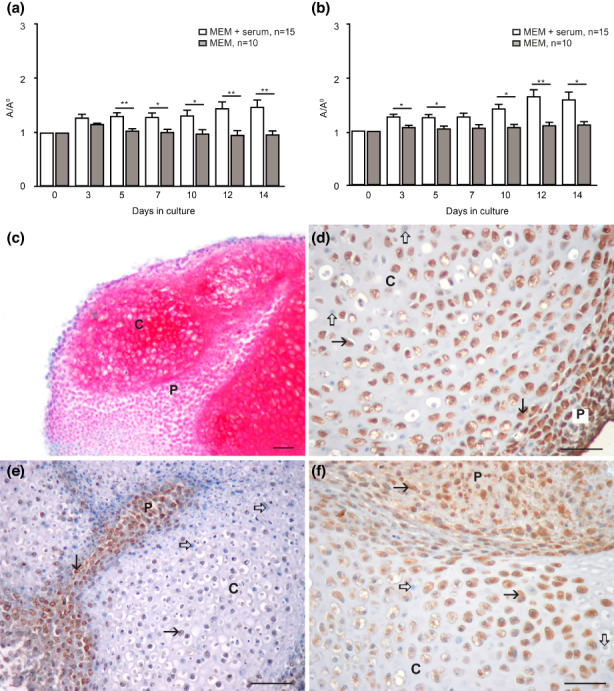
Development of limb buds in vitro. (a) Overall growth of front‐limb buds. Mean ± SEM, Student's *t*‐test. A/A0 = explant area. (b) Overall growth of hind limb buds. A/A0 = explant area. Student's *t*‐test; *p < 0.05, **p < 0.01. (c) Differentiation of cartilage in a front‐limb bud cultivated in the serum‐free medium, Safranin O stain. (d) 8‐OHdG expression in a front‐limb bud cultivated in the serum‐supplemented medium. 8‐OHdG signal (arrow), negative inner control (hollow arrow). IHC, DAB, contrasted with haematoxylin. (e) Nitrotyrosine in a front‐limb bud cultivated in the serum‐supplemented medium. Nitrotyrosine signal (arrow), negative inner control (hollow arrow). IHC, DAB, contrasted with haematoxylin. IHC, DAB, contrasted with haematoxylin. (f) 8‐OHdG in a hind‐limb bud cultivated in the serum‐free medium. 8‐OHdG signal (arrow), negative inner control (hollow arrow). IHC, DAB, contrasted with haematoxylin. DAB, contrasted with haematoxylin

We first compared the growth of GD 14 front and hind‐limb buds in the simple chemically defined protein‐free medium (MEM) and metabolically richer serum‐supplemented MEM. During cultivation in the chemically defined MEM alone, the growth of whole explants was continuously lower than in the serum‐supplemented medium. This difference started on the 3rd day of culture, and it was statistically significant except in FLBs on the 3rd day and HLBs on the 7th day of the culture period (Figure 1a,b).

After the 14 days of culture, we confirmed that cartilage was terminally differentiated in both serum‐supplemented and serum‐free medium (Figure 1c) as published earlier (Mužić Radović et al., 2022). At the end of the culture period, we also assessed the expression of reactive oxygen and nitrogen species (RONS) that are the markers of oxidative/nitrosative stress and may affect intracellular signalling (Weidinger & Kozlov, 2015). The results showed that the marker of oxidative stress 8‐Hydroxy‐2′‐deoxyguanosine (8‐OHdG) was expressed in nuclei of the cartilage and perichondrial cells differentiated in both serum‐supplemented and serum‐free mediums. However, the cytoplasmic marker of nitrosative stress (nitrotyrosine) was expressed only in serum‐supplemented limb bud cultures and was absent from the serum‐free limb bud cultures (Figure 1).

**FIGURE 2:**
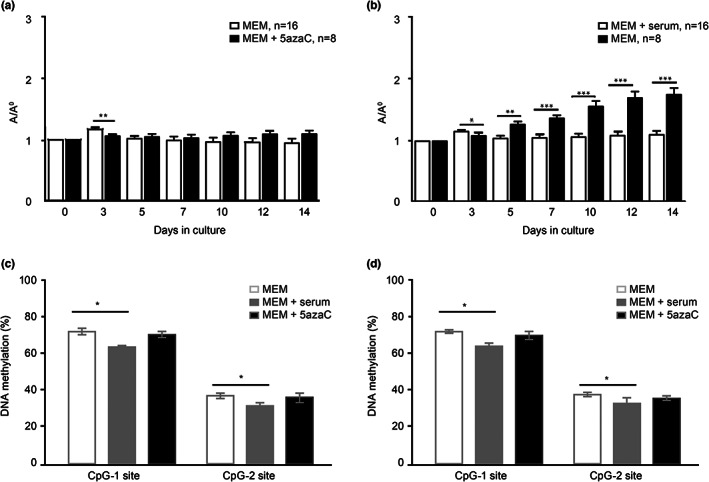
Overall growth and global DNA methylation of limb buds for two weeks in vitro in the minimum essential medium (MEM) and MEM with 5 μM 5‐azacytidine (mean ± SEM). (a) Front limb buds; (b) hind limb buds. A/A0 = explant area at individual days of culture/explant area at the beginning of culture, Student's *t*‐test, *p < 0.05, **p < 0.005, ***p < 0.0001. (c) Front limb and (d) hind limb DNA methylation of rat ID element. Six samples per group, Mann–Whitney *U*‐test

Additionally, we compared the growth of front and hind‐limb buds in the simple chemically defined protein‐free medium (MEM) and MEM with the addition of 5‐azacytidine (5azaC), a DNA‐hypomethylating agent that is known to diminish the growth of limb buds in the serum‐supplemented culture (Muzic et al., 2013). As expected, 5azaC significantly inhibited the overall growth of LBs, as shown on the third day of culture. On the contrary, during the rest of the 14 days culture period, adding 5azaC to MEM improved growth compared to MEM‐only, but this was statistically significant only for HLBs (Figure 2a,b). Furthermore, no statistically significant decrease in the global DNA methylation was found on the last day of culture in cultures treated with 5azaC. However, the global methylation in serum‐supplemented cultures was significantly lower than in serum‐free cultures (Figure 2c,d).

### 3.2 Differentiation in transplants of limb buds pre‐cultivated in the simple protein‐free chemically defined medium

**FIGURE 3:**
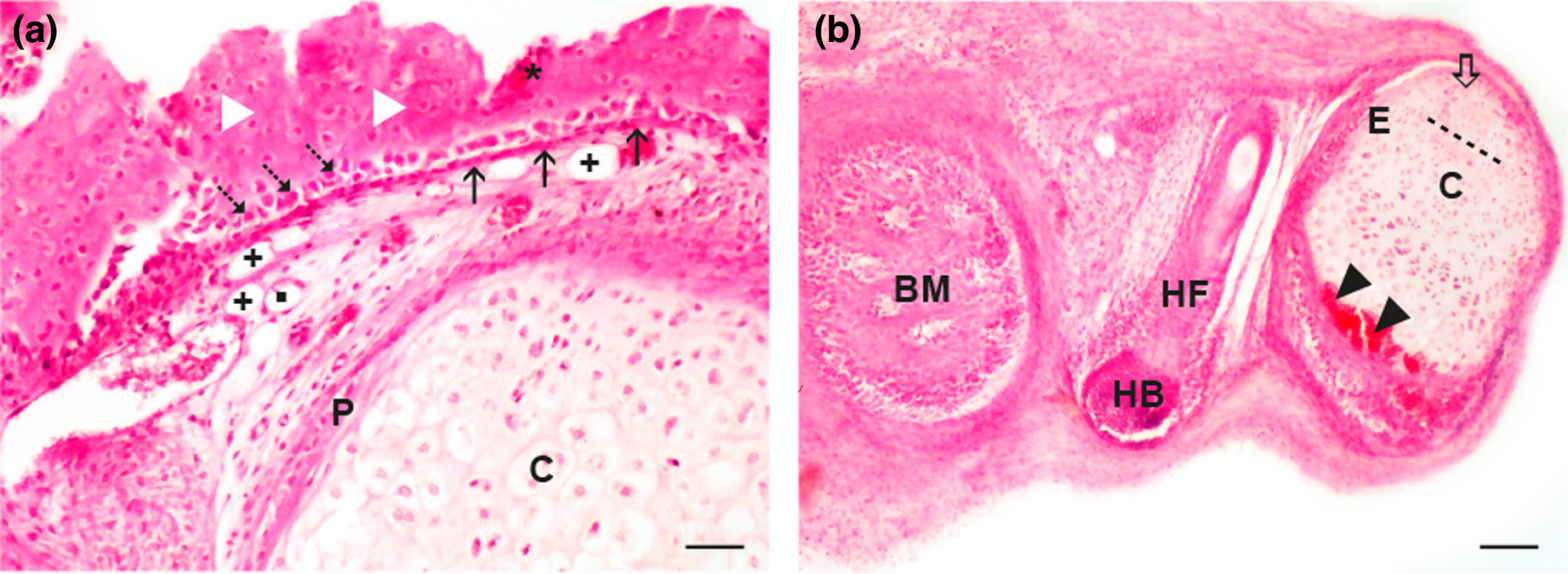
Osteogenesis in transplants. (a) Transmembranous osteogenesis and cartilage in a hind limb bud transplant precultivated in a chemically defined protein‐free medium. Osteocytes (white triangle); osteoclasts (asterisk); *stratum fibrosum* (thin arrow); *stratum germinativum* (dashed arrow); perichondrium (P); chondrocytes in lacunae (C); fat cell (cross); capillary (rectangle). (b) Enchondral ossification and a hair bulb in a transplant of a front limb bud pre‐cultivated in a chemically defined protein‐free medium. Hair follicle (HF); hair bulb (HB); enchondral ossification (E); resting zone (thick arrow); proliferative zone (dashed line); zone of hypertrophic cartilage (C); ossification zone (arrowhead); bone marrow (BM). Scale bar 100 μm, haematoxylin–eosin stain

A significant difference in the growth of cultivated limb buds was the most consistent between those cultivated in the chemically defined medium without any supplementation and those cultivated with serum supplementation (Figure 1). To assess their remaining differentiation potential, we transplanted samples from these groups in vivo. Beneath the renal capsule, transplants developed into structures with a mixture of tissues that did not retain the exact organotypic arrangement of the developing limb buds. Nevertheless, differentiation progressed into various tissues typical for the advanced development within the limb buds that have never been found in cultivated‐only limb buds. In all transplants, two types of osteogenesis were visible (Figure 3). In transmembranous osteogenesis (desmal ossification), periosteal layers stratum fibrosum and stratum germinativum containing typical cuboidal osteoblasts were found. In the abundant osteoid, osteocytes were typically situated in lacunae, while osteoclasts were found at the top of the differentiated bone (Figure 3a). Enchondral ossification with typical zones (resting, proliferative, hypertrophic, and mineralization) was also found (Figure 3b).

**FIGURE 4:**
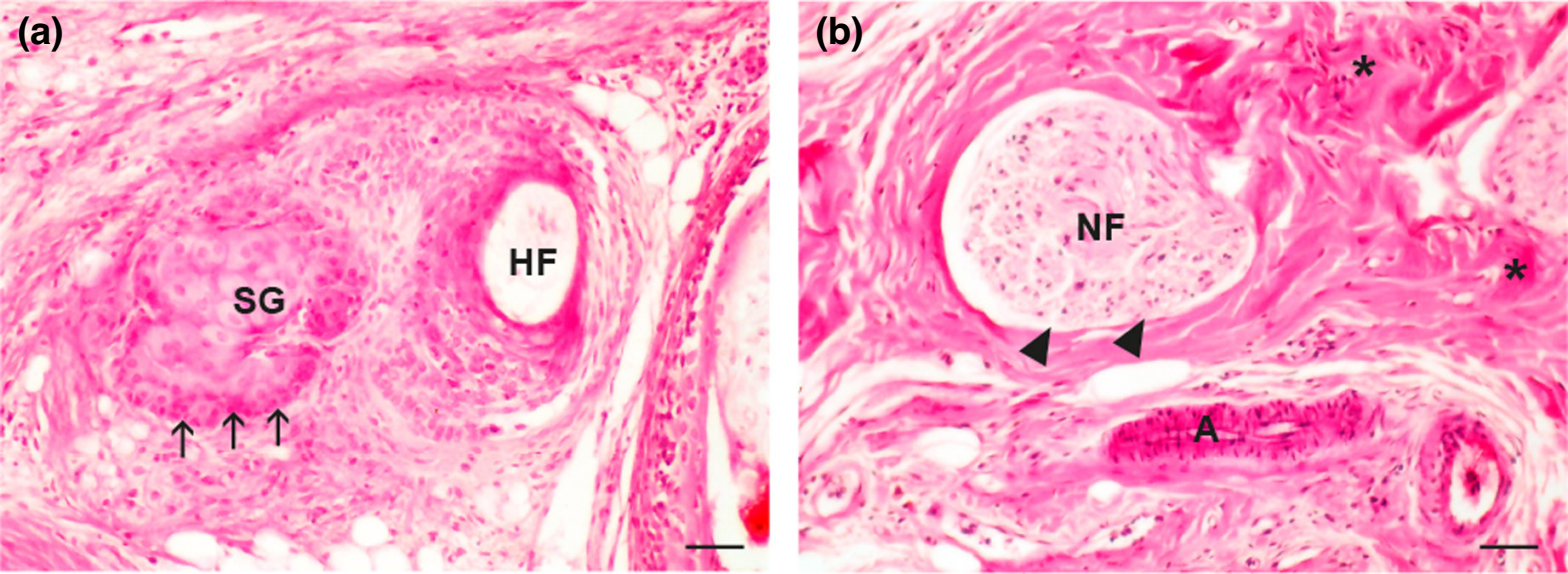
Skin appendages and nerves in transplants. (a) Skin appendages in a transplant of a front limb bud pre‐cultivated in a chemically defined protein‐free medium. Hair follicle (HF); sebaceous gland (SG); sebaceous gland stem cells (arrows). (b) Nerve, blood vessels, and fibrous tissue in a transplant of a front limb bud pre‐cultivated in a serum‐supplemented medium. A bundle of nerve fibres (NF), *perineurium* (arrowheads), collagen fibres (asterisk), artery (a). Scale bar 100 μm, haematoxylin–eosin stain

**FIGURE 5:**
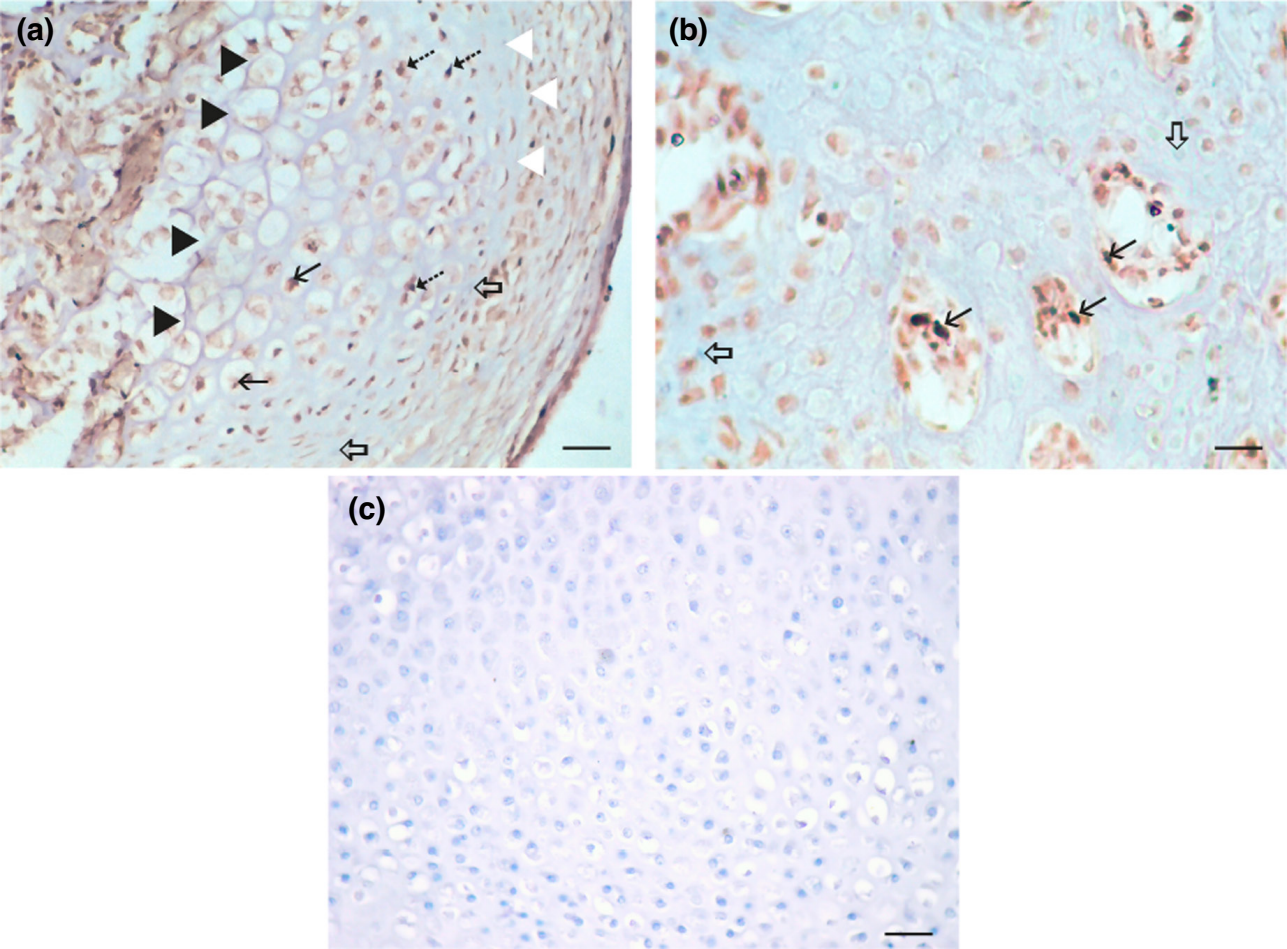
Proliferating cell nuclear antigen (PCNA) expression in transplants. (a) Enchondral ossification in a hind limb transplant precultivated in a chemically defined protein‐free medium with 5 μM 5‐azacytidine. Resting zone (white arrowhead); proliferative zone (dashed arrow); calcification zone (black arrowhead), PCNA signal (thick arrow), negative inner control (hollow arrow). IHC, DAB, contrasted with haematoxylin. (b) Bone marrow in a transplant of hind limb bud precultivated in a chemically defined protein‐free medium with 5 μM 5‐azacytidine. PCNA expression in blood cell precursors (arrows), negative inner control (hollow arrow). (c) Negative control. IHC, DAB, haematoxylin counterstain

Moreover, bone marrow, mature connective tissue, fat cells, and blood vessels (Figure 3) were also detected in all transplants (Figure 3). Differentiation of surface epithelium also progressed, and skin appendages (hair follicles and sebaceous glands) were consistently found (Figure 3b and 4a). On the other hand, a bundle of nerve fibres with a perineurium was found only in transplants pre‐cultivated in the serum‐supplemented culture (Figure 4b). In transplants, expression of the proliferating cell nuclear antigen (PCNA) was detected in cells with the remaining proliferation potential of various tissues such as the cartilage, periosteum, bone marrow, blood vessel‐endothelium, and hair follicle (Figure 5).

Therefore, in the richer in vivo environment, differentiation of pre‐cultivated explants progressed regardless of the pre‐cultivation medium except for the neural tissue that could not differentiate without the serum supplementation during the cultivation period. It must be emphasized that pre‐cultivation in the simple chemically defined serum‐ and protein‐free medium enabled both transmembranous and enchondral ossification in transplants.

## 4 DISCUSSION

The presented results show that cartilage originating from the early rat limb buds pre‐cultivated in a serum‐ and protein‐free medium (Mužić Radović et al., 2022) preserves the full potential for osteogenesis in vivo. This finding is in accordance with the results of other authors showing that the self‐organizing and self‐patterning capacity of the mouse embryonic cartilage is retained even when subjected to cellular dissociation (Fernando et al., 2018). In contrast to some experiments with dissociated cells, where necessary hydrogel encapsulation heavily influenced bone‐forming potential in the subcutaneous ectopic assay in vivo (Verbeeck et al., 2019), we used natural encapsulation by the kidney capsule. The latter technique has been successfully used before for direct transplantation of parts of rodent embryos, epiblast, three germ‐layer gastrulating embryos‐proper, epiglottis, and mandible which always continued their development in such a secluded and well‐vascularized space (Bulic‐Jakus et al., 2016; Marinovic‐Kulisic et al., 2011; Radujkovic et al., 2020). It was described recently that subcapsular kidney space was not optimal for differentiation of cartilage engineered from human embryonic stem cells in specific chemically defined serum‐free conditions because it gave rise also to the bone in contrast to the subcutaneous transplantation (Yang et al., 2016). Therefore, transplantation to the subcapsular kidney space and the subcutaneous space can both reveal the full developmental potential of transplanted tissue. Similarly, as found in our research with limb buds, the growth of the rat gastrulating embryos in vitro was significantly enhanced in the serum‐supplemented medium compared to the protein‐free medium. However, both culture conditions had the same impact on bone and skin appendages differentiation in transplants (Belovari et al., 2001).

To explain enhanced growth in vitro, as found in LB explants grown with 5azaC in the protein‐free medium, we found no significant effect of the DNA demethylating 5azaC on the global DNA methylation. Previously we found a rise in global DNA methylation in limb buds cultivated serum‐free that was higher than in limb buds cultivated with serum supplementation (Mužić Radović et al., 2022), so this effect could hide the 5azaC‐demethylating effect. Furthermore, we have confirmed that histologically, cartilage differentiated equally well in both simple serum‐free and serum‐supplemented media culture conditions in vitro. However, expression of the oxidative stress markers 8‐OHdG and nitrotyrosine, both found in the serum‐supplemented medium, might point to cartilage degeneration such as found in ageing or osteoarthritis (Chen et al., 2017; Loeser et al., 2002; Wang et al., 2013; Zahan et al., 2020).

Importantly, there is a difference in expression of the nitrosative stress marker, nitrotyrosine, that was absent from the serum‐free cultivated LB cartilage compared to serum supplementation‐cultivated LB cartilage. Knowing that protein‐free conditions might elicit oxidative stress and suppress cell division (Burdon et al., 1989), the lack of the nitrosative marker points to a certain effect on the signalling pathway that may also be changed by the reactive nitrogen and reactive oxidizing species (Weidinger & Kozlov, 2015). These results are worth investigating further because RONS expression was associated with DNA demethylation (Sobočan et al., 2019).

We did not assess any impact of the significantly enhanced overall growth in the serum‐supplemented medium throughout the culture period on the quality of differentiation in transplants. This finding contrasts the cultivation of cartilage‐derived dissociated cells where FGF2 was used in the cell expansion protocol enriched for skeletal stem cell markers, but a significant loss of in vivo osteogenesis was observed (Verbeeck et al., 2019). To explain this difference, we must first remember that our LB and gastrulating embryo in vitro systems are a part of complex solid tissue which partly retain original tissue interactions that are entirely lost from the dissociated cartilage. Next, apotransferrin or bovine serum albumin, as the only protein supplements, significantly enhanced the growth of the gastrulating rat embryo in vitro, although not to the level found in serum‐supplemented cultures (Skreb et al., 1993). These proteins also significantly enhanced the incidence of bone in subsequent transplants compared to those pre‐cultivated with or without serum (Belovari et al., 2001).

Although the used culture conditions had no impact on the skin‐appendage differentiation, the quality or type of osteogenesis (transmembranous and enchondral), or bone marrow differentiation, neural differentiation was observed only in transplants originating from the serum‐supplemented LB cultures. This result follows our previous research on well‐differentiated neural tissue derivatives developing with a significantly lower incidence and no difference in the differentiation of skin appendages from the gastrulating embryo pre‐cultivated in the protein‐free medium where the growth was absent. However, cultivation with transferrin or BSA as the only proteins enhanced growth in vitro and subsequent differentiation of neural derivatives in transplants (Belovari et al., 2001; Bulic‐Jakus et al., 2001). Therefore, the optimal levels of growth in culture conditions do not seem to be directly associated with the potential of mesenchymal derivatives such as cartilage, bone, or the ectodermal derivate epidermis to differentiate in vivo subsequently. The preservation of their competence might be more important (Gilbert, 2000). The competence may be changed or lost during complex experimental procedures that may influence the permissiveness of the microenvironment in different ways. Deciphering the influence of various microenvironments showed that even specific physicochemical properties, e.g., scaffolding materials and artificial extracellular matrices are essential for osteogenic differentiation in vitro and in vivo. Namely, graphene‐derivative nanomaterials can change various stem cells' growth and differentiation potential. Graphene‐oxide may promote osteogenesis of pluripotent human mesenchymal stem cells (MESCs) through the ability to act as a preconcentration platform for osteogenic inducer dexamethasone and β‐glycerolphosphate or enhancement of BMP‐SMAD1/5 signalling pathway (Ikram et al., 2022; Lee et al., 2011; Li et al., 2021). Lately, biodegradable scaffolds enriched with MESC have been successfully used for in situ bone regeneration in the mouse (Du et al., 2021).

We conclude that the simple chemically defined serum‐ and protein‐free medium (Eagle's MEM), used for pre‐cultivation of mammalian limb buds in vitro before their transplantation in vivo, is permissive for enchondral and transmembranous osteogenesis and differentiation of skin appendages but it is non‐permissive for neural tissue differentiation. These results are important for a better understanding of limb development and in vitro/in vivo regenerative medicine strategies.

## CONFLICT OF INTEREST

The authors have no conflicts of interest to declare.

## Data Availability

The data that support the findings of this study are available from the corresponding author, M.H.P. upon reasonable request.
